# A Histopathological Exploration of the *Madurella mycetomatis* Grain

**DOI:** 10.1371/journal.pone.0057774

**Published:** 2013-03-06

**Authors:** Anahid Izzat Ibrahim, Ahmed Mohammed El Hassan, Ahmed Fahal, Wendy W. van de Sande

**Affiliations:** 1 Faculty of Medical Laboratory Sciences - University of Khartoum, Khartoum, Sudan; 2 Mycetoma Research Center, University of Khartoum, Sudan, Khartoum, Sudan; 3 Erasmus MC, Department of Medical Microbiology & Infectious Diseases, Rotterdam, The Netherlands; University of Illinois at Chicago, United States of America

## Abstract

Although the *Madurella mycetomatis* grains seem to interfere with the host defense mechanisms and impede the antifungal drugs penetration, yet their histological features are not fully known and hence this study was set out to determine that. The study included 80 patients with confirmed *M. mycetomatis* eumycetoma. After informed written consent, surgical biopsies were obtained from the excised tissues during the patients’ surgical treatment. All sections were stained with haematoxylin and eosin, Grocott’s hexamine silver, Periodic Acid-Schiff’s, Masson-Fontana, Perl’s Prussian Blue, Von-kossa’s, Formalin Inducing Fluorescence and Schmorl’s stains. Modified bleaching technique was used. The concentrations of Zinc, Copper, Calcium, Iron, Lead, Cobalt and Nickel were determined by Atomic Absorption Spectrophotometer. The *M. Mycetomatis* grains appeared to consist of lipid, protein and melanin. The melanin was located on the hyphal wall as thick layers. The Zinc, Copper and Calcium concentrations in the grains were four, six, and sixteen folds higher than in normal tissue respectively, the other metals were found in the same concentrations as in normal tissue. In the grains, calcium was located in the melanin vicinity. From this study, it can be concluded that, the grains contain melanin, heavy metals, proteins, lipids and they contribute in the formation of the grain cement matrix. These elements seem to contribute in the organism pathogenicity and might impede the penetration of various anti-fungal agents.

## Introduction

Mycetoma is a chronic, subcutaneous granulomatous, progressive and destructive inflammatory disease. It is caused by true fungi or by certain bacteria and hence it is classified as eumycetoma and actinomycetoma respectively [1]. The triad of painless subcutaneous mass, multiple sinuses and sero-purulent discharge containing grains is characteristic of mycetoma. It may spread to involve the skin and the deep structures including bone resulting in destruction, deformity and loss of function and in many cases it is difficult to treat and sometimes mycetoma may be fatal [2], [3].

*M. mycetomatis* is the most common causative organism of eumycetoma worldwide and it is characterized by the formation of black grains in the tissue. These grains consist of densely packed fungal mycelia embedded in a hard and brown-black cement material [4]. Histologically the grain appears rounded, oval, or trilobed and has a medulla and more compact cortex. Two types of grains were described: the filamentous and the vesicular [4]. The chemical composition of these grains is not fully understood.

It is to be suspected that some of the main components of the fungal grain are probably organic substances such as carbohydrates, lipids, proteins and nucleic acids. These components are in direct contact with the host and may therefore play an important role in the fungal pathogenicity and possibly in resistance to various antifungal agents [5], [6], [7], [8]. In general, fungal melanin’s are known to play important roles in protecting the fungus against UV light when the fungus is in the soil and from the host immune responses and antifungal drugs when it is in the host [9], [10], [11]. Another property of fungal melanins is that, they can bind to heavy metals. The fungal melanin’s carboxyl, hydroxyl, phenolic and amino groups bind to the heavy metals and induce most of the host damage [12].

Still there are controversies regarding eumycetoma pathogenesis, but it is certain that the grain plays an important role. Since the composition of the fungal grain in mycetoma is hardly studied, this prospective study was undertaken to determine the nature of *M. mycetomatis* grains and to measure the concentrations of the heavy metals and organic substances within the grains. When the exact nature of the mycetoma grains becomes known, new therapies can be developed which target the mycetoma grains themself and which hopefully will finally result in a proper therapy for this mutilating disease.

## Materials and Methods

This is a prospective descriptive hospital based study which was conducted at the Mycetoma Research Centre, Soba University Hospital, University of Khartoum, Khartoum-Sudan after obtain ethical clearance from Soba University Ethical Committee.

### Section Material and Histological Staining

After written informed consent, eighty surgical biopsies containing black grains were randomly obtained from the excised tissues during the patients’ surgical treatment. All patients had confirmed *M. mycetomatis* eumycetoma. Five 1×1 cm subcutaneous biopsies were obtained from five patients undergoing surgery for other conditions. The biopsy sites were the foot and hand. These were used as controls and were treated in the same manner as the patients’ biopsies. Sections from a lymph node metastatic melanoma and from normal liver biopsy were included as a positive control and negative control respectively for melanin staining.

The surgical biopsies were fixed in 10% formal-saline for 24 hours and paraffin blocks were prepared. All sections were stained with haematoxylin and eosin (H&E), Grocott’s hexamine silver, Periodic Acid-Schiff’s (PAS), Masson-Fontana., Formalin Inducing Fluorescence (FIF) to detect melanin. The Von-kossa’s stain was used to detect calcium ions. To detect ferric iron, Perl’s Prussian Blue stain (PPB) was used.

The modified bleaching technique was used to determine the presence of melanin in the grains. In short, *M. mycetomatis* pigment was bleached in 1% potassium permanganate solution for three hours, treated in oxalic acid for one minute then dehydrated in absolute ethanol. It was then cleared by xylene and mounted in Dystrene plastizier and xylene.

### Biochemical Analysis

Grains were isolated randomly from *M. mycetomatis* tissue biopsies and placed in sterile containers containing normal saline with chloramphenicol, then crushed, dissolved in distilled water and the following biochemical tests were done; Benedict’s test for sugar, Biuret’s test for protein and Sudan III test for lipids.

### Atomic Absorption Spectrophotometer (AAS) Analysis

The concentrations of Copper (Cu), Zinc (Zn), Calcium (Ca), Iron (Fe), Lead (Pb), Cobalt (Co) and Nickel (Ni) were determined by the flame and furnace AAS using the ashing technique as described by Sadik, (2001) [13] in five *M. mycetomatis* grains samples, taken randomly and in the normal five subcutaneous biopsies; the controls.

The grains and the normal subcutaneous tissue were dried at 120°C three times for 3 hours each. The material (ashes) was left to cool to room temperature. The residues of the samples were dissolved in 5 ml of 5 M HCl and few drops of HNO3 were added to avoid dryness by evaporation of HCL when the samples were left in the water bath at boiling point. The residues were re-dissolved in 5 ml of 5 M HNO3 and left to cool. Then the dissolved residues were filtered into a 25 ml volumetric flask and the volume was made up to 25 ml with distilled water. Finally, the samples were read in an atomic absorption spectrophotometer (GFA-EX7, Shimadza Corporation, USA) by Furnace AAS for Cu, Zn, Pb, Ni and Cd and by Flame AAS for Cu, Zn, Ca and Fe.

### Grain Solubility Property

In order to determine the solubility of the *M. mycetomatis* pigment, two grams of isolated *M. mycetomatis* grains were placed in several clean glass tubes. Five milliliter of normal Sodium hydroxide (NaOH), concentrated Potassium hydroxide (KOH), concentrated hydrochloric acid (HCL)(1∶4), diluted HCL (1∶10), HCL with ethanol, Chloroform (organic solvent) and three inorganic solvents; Acetone, Butyl alcohol and Isopropyl alcohol were added to each tube separately. After overnight incubation the change in solvent colour was determined. The change of solvent colour indicated melanin solubility.

### Statistical Analysis

The data was managed by SPSS computer programme version 10 (SPSS –USA) and t-student test was used for means comparisons.

## Results

This study included 80 patients with confirmed eumycetoma due to *M. mycetomatis*. Seventy of the patients were males, their ages ranged was between two and 50 years with a mean age of 20.5±2.2 years. The disease duration ranged between six months and 11 years with a mean duration of 5.4±2.1 years. Thirty patients had massive disease and 40 patients had recurrent mycetoma.

### Grain Morphology and Biochemical Composition of the Grain

**Figure 1:**
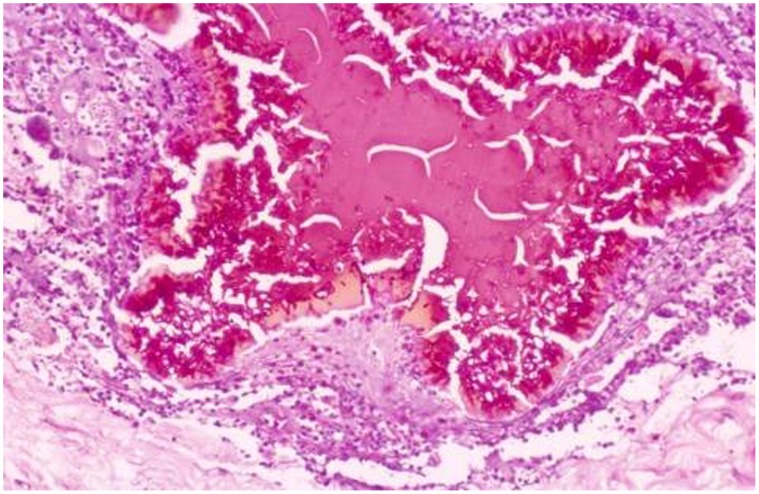
The photomicrography shows a vesicular type of grain. The hyphae are mainly in the periphery and are swollen. The centre of the grain consists of cement substance (H&E×40).

In the patients group, both filamentous and vesicular grain types were observed in H&E stain, all grains contained a cement material which was seen as a homogenously stained pink material in which individual hyphae were embedded. Different grains sizes were noted and that ranged between 0.5–3 mm. (Fig. 1).

To study the biochemical composition of the grains, some universal biochemical tests were performed on the grain and the cultured mycelia. The Benedict’s test for sugars demonstration was negative in all the grains and cultured mycelia samples although the test is not accurate. Both Biurt’s test for protein and Sudan ΙΙΙ test for fat were positive with both the grains and cultured mycelia. This may indicate that fungal grain consisted mainly of fat and proteins.

### Melanin is Part of the M. mycetomatis Grain

**Figure 2:**
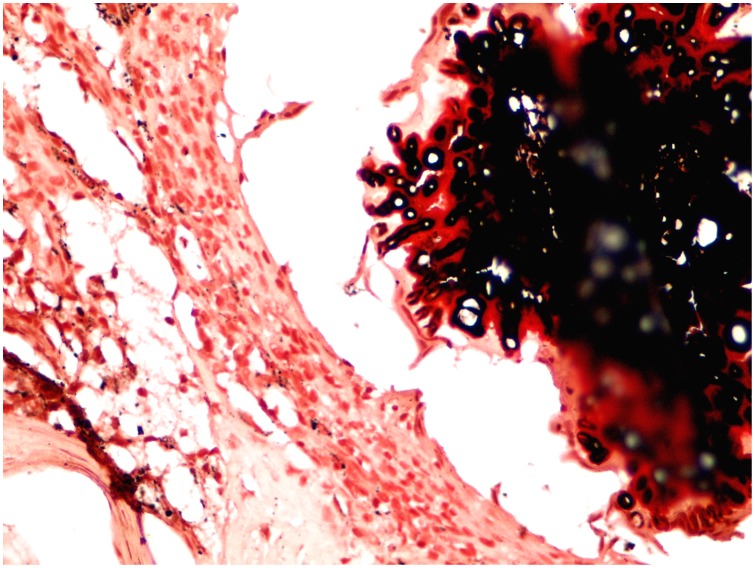
The photomicrography shows melanin in the grain. The hyphae at the periphery of the grain are covered with melanin (Masson-Fontana stain for melanin ×40).

**Figure 3:**
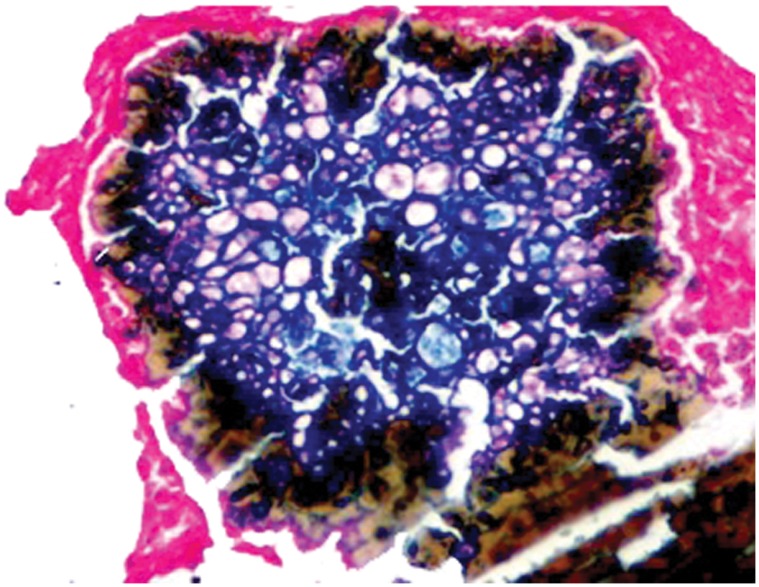
The photomicrography shows cements substance and hyphae staining a blue colour with Schmorl’s stain for melanin (Schmorl’s stain ×40).

Since *M. mycetomatis* grains are black coloured many researchers considered the grain colour originating from melanin. Here we used different stains to detect the presence of melanin in the grains and the surrounding tissue. Masson-Fontana stain demonstrated the presence of melanin inside the *M. mycetomatis* grain and it was shown as brown-black, similarly to our control of a malignant melanoma melanin section. The pigment was seen in the mycelial cell wall as a thick layer, some was present as a diffused pigment in the cement matrix or engulfed by macrophages in the grain surrounding tissue. (Fig. 2). Furthermore, the *M. mycetomatis* pigment stained blue with the Schmorl’s stain indicating the presence of melanin. With this stain the melanin appeared to be found mainly in the fungal cell walls and inside the macrophages. (Fig. 3) The cement matrix was stained yellow-brown color indicating the absence of melanin The *M. mycetomatis* pigment, with FIF stain, had weak yellow fluorescence. Potassium permanganate had bleached the pigment confirming it was melanin in nature.

### The *M. mycetomatis* Pigment could be Dissolved in both Acid and Alkaline Solutions

The *M. mycetomatis* pigment was soluble in HCL, NaOH and KOH, however, the pigment was insoluble in both the organic solvent (Chloroform), and the inorganic solvents (Acetone, Butyl alcohol, Isopropyl alcohol) and diluted HCL.

### High Concentrations of Cu, Zn and Ca were Bound to the Mycetoma Grain

**Figure 4:**
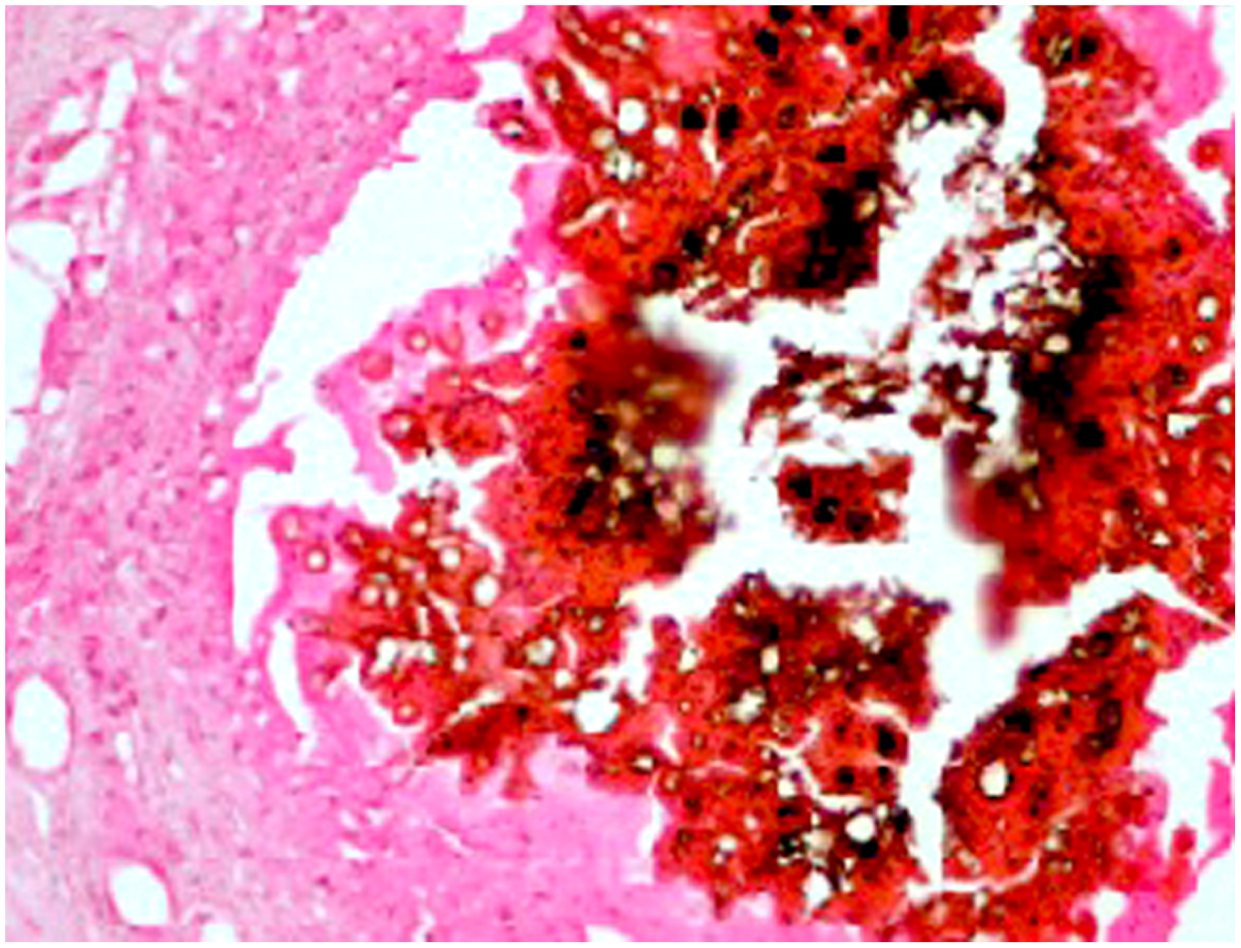
The photomicrography shows the hyphae and cement substances are positive for calcium (Von Kossa stain ×40).

Since it was already known that, ions and heavy metals bind to melanin, both staining techniques and atomic absorption spectrometry were used to determine the presence of these ions in the mycetoma grain. The staining techniques showed that certain ions were present in the grain, while others were not. The Perl’s Prussian blue reaction for iron was negative in the *M. mycetomatis* grain but it was blue positive in the surrounding inflammatory tissue. Calcium ions which stained black-brown in colour with the Von-kossa stain was positive. Calcium ions were demonstrated in the mycelial cell wall, at the melanin sites and in the surrounding tissue. (Fig. 4).

Atomic Absorption Spectrophotometry (AAS) was used to determine the concentrations of several metals in the grains and the surrounding tissue and the controls. The concentrations of Cu, Zn, and Ca in the grains were four, six and sixteen times higher respectively than in normal control tissues (p<0.05). There were no significant differences in the Fe, Ni and Co concentrations between the patients and control, (p>0.05). (Table 1).

**Table 1:** The mean values of the heavy metals concentrations in the patients’ biopsies & the control.

Metals	Normal tissue(Mean/ppm)	Grains(Mean/ppm)
Cu	0.04±0.018	2.7±1.15
Zn	0.34±0.1	7.6±4.8
Ca	1.20±0.15	52.1±17.6
Fe	4402.9±336.7	6795.4±2753.9
Pb	00	00
Ni	00	00
Co	00	00

## Discussion

One of the main characteristics of mycetoma is that, the causative agent organises and protects itself in grains. The grains are different in colour, size and consistency, according to causative agent. Furthermore, some causative agents produce a cement material within the grains while others do not. *M. mycetomatis* produces large black grains with cement material, *Streptomyces somaliens*is produces medium size yellow grains with cement material while *Actinomadura pelletierii* grains are tiny red in colour with no cement material [1], [3], [4].

Although the *M. mycetomatis* grains were described in 1905 by Brumpt, [14] yet the exact nature of these grains are still not well known. Hence this prospective study was set out to determine the grain composition to have clear inside on the grains nature. In this study, it was demonstrated that, the fungal grain contains lipids, proteins, melanin and elevated concentrations of Cu, Zn, and Ca.

However, the nature of lipids and proteins are not specifically known in this study and further studies are needed to determine their nature, but in general proteins and lipids are known to contribute for the virulence of some fungi [8], [15]. The proteins can be of host and fungal origin. In many fungal infections it has been demonstrated that, proteases secreted by fungi can interfere with the host tissue responses and can elicit an immune reaction as well. *Aspergillus fumigatus* for instance uses certain proteins to avoid the host complement attacks. It is interesting to note no sugar in the grain was detected in this study and this contrasts with that reported by Van de Sande and colleagues, this can be due the fact that, the test used here was not a sensitive enough to detect that [16].

The various stains used in this study, demonstrated the presence of melanin in the fungal cell walls as thick layers, diffused in the cement matrix or engulfed by macrophages. *M. mycetomatis* most probably uses this melanin as a protective mechanism which enhances the fungal survival. This is in line with that reported by Behnsen [15] who suggested that, the fungal melanin protects the fungi from various host defense mechanisms such as the hydrolytic enzymes, free radicals, redox buffering, and from the host defensive proteins such as antibodies and complement by binding with melanin or by polymerized melanin covering cell wall linkages.

The *M. mycetomatis* melanin also acts as an anti-oxidant agent and it resists the antifungal effects of Ketoconazole and Itraconazole. This is documented by the fact that, adding isolated melanin to the culture medium results in elevation of the minimum inhibitory concentrations of these antifungal agents [16].

Many reports demonstrated the great affinity of the heavy metals to melanin [17], [18], [19]. The fungal melanin’s carboxyl, hydroxyl, phenolic and amino groups bind to the heavy metals and induce most of the host damage [17], [18]. In this study, the heavy metals were found in higher concentrations in mycetoma lesions compared with normal tissue and this is in accordance the mentioned observations. The melanin-metals complex is a fungal protective mechanism as it acts as physiological redox buffer, a sink for harmful unpaired electrons and provides the cell walls structural rigidity and store water and ions to prevent desiccation [20].

In this study, the *M. Mycetomatis* pigment proved to be soluble in both strong alkalines and acids but it was insoluble in most organic and inorganic solvents. This contrasts with human melanins, mainly eumelanin, which is soluble only in strong alkalines such as NaOH [11]. However, the clinical significance of this property is not clear.

In general, fungi usually consume metals from the surrounding environment, [20] and most probably the grain high metal concentrations demonstrated in this study were obtained from the host tissue. The fungal acquirement of these metals, is most probably are obtained locally; from blood and the surrounding tissue. The concentrations are small in amounts, since none of the patients with mycetoma showed obvious clinical evidence of heavy metal deficiency. However, measurement of the serum concentrations of these metals is needed to verify this.

In this study, calcium ions, were present in high concentrations in the fungal cell wall in close proximity to the melanin sites which may indicate that calcium is bound directly to the melanin. The presence of the calcium in this locality may give the grain its hardness and contribute the *M. mycetomatis* cement substance. This is in line with that reported by Findlay & Vismer who showed that, grains sclerosis, and melanization are essential for the cement substance formation [21], [22].

From this study, we may suggest that other heavy metals may contribute to the formation of the cement substance as well. Findlay in 1977 used the Atomic absorption and spark source mass spectrophotometry to study the *M. mycetomat*is grain and reported a slight increase in the concentrations of mineral constituents and no increase of calcium in mycetoma lesions when compared to normal tissues [22]. This contrasts with our results and the method used in that study probably was not sensitive to detect that, furthermore the number of biopsies included in this study was more, which gives more credibility to the present study.

In this study, high copper concentration inside the fungal grain was detected. In saprophytes, it was demonstrated that, DHN-melanin is produced in response to the presence of copper [12]. Copper has an essential role in the connective tissue formation, mainly the collagen fibers [23]. Although the causation of fibrous capsule formation around the mycetoma lesion may be multi- factorial, copper may be one of those factors.

In conclusion, in this study it was demonstrated that, *M. mycetomatis* grain contains melanin, heavy metals, proteins and lipids. These elements most probably contribute to the formation of cement matrix, in which the mycelia are embedded and can impede the penetration of various anti-fungal agents and this is in line with that reported by Roderick previously [24].

Therapeutic modalities that can target and destroy the grains melanin and matrix material are needed. These will expose the fungus to both the host immune responses and the effects of the antifungal agents which eventually improve the management, outcome and prognosis of mycetoma which is still a mutilating disease in many tropical and subtropical regions.
